# Insight into the direct interaction of Na^+^ with NhaA and mechanistic implications

**DOI:** 10.1038/s41598-021-86318-8

**Published:** 2021-03-29

**Authors:** Matthias Quick, Manish Dwivedi, Etana Padan

**Affiliations:** 1 Department of Psychiatry and Center for Molecular Recognition, Columbia University Vagelos College of Physicians and Surgeons, and New York State Psychiatric Institute, New York, NY 10032 USA; 2 Department of Biological Chemistry, Alexander Silberman Institute of Life Sciences, The Hebrew University of Jerusalem, Edmond J. Safra Campus, Givat Ram, 91904 Jerusalem, Israel; 3 Present Address: Amity Institute of Biotechnology, Amity University Uttar Pradesh, Lucknow, 226028 India

**Keywords:** Biochemistry, Proteins, Membrane proteins, Nanoparticles

## Abstract

Na^+^/H^+^ antiporters comprise a family of membrane proteins evolutionarily conserved in all kingdoms of life that are essential in cellular ion homeostasis. While several human homologues have long been drug targets, NhaA of *Escherichia coli* has become the paradigm for this class of secondary active transporters as NhaA crystals provided insight in the structure of this molecular machine. However, structural data revealing the composition of the binding site for Na^+^ (or its surrogate Li^+^) is missing, representing a bottleneck in our understanding of the correlation between the structure and function of NhaA. Here, by adapting the scintillation proximity assay (SPA) for direct determination of Na^+^ binding to NhaA, we revealed that (i) NhaA is well adapted as the main antiporter for Na^+^ homeostasis in *Escherichia coli* and possibly in other bacteria as the cytoplasmic Na^+^ concentration is similar to the Na^+^ binding affinity of NhaA, (ii) experimental conditions affect NhaA-mediated cation binding, (iii) in addition to Na^+^ and Li^+^, the halide Tl^+^ interacts with NhaA, (iv) whereas acidic pH inhibits maximum binding of Na^+^ to NhaA, partial Na^+^ binding by NhaA is independent of the pH, an important novel insight into the effect of pH on NhaA cation binding.

## Introduction

Na^+^/H^+^ antiporters are present in membranes of nearly all eukaryotic and prokaryotic cells and organelles^1^ where they maintain the homeostasis of pH, Na^+^ concentration, and cell volume^2^. Prokaryotic Na^+^/H^+^ antiporters contribute to the understanding of their human homologues involved in diseases. For example, NHE1, which is implicated in heart failure, has long been used as a drug target^3^. Certain other human homologues are potential drug targets because they are involved in essential hypertension^4^, *Diabetes mellitus*^5^, and cancer^6^. Furthermore, Na^+^/H^+^ antiporters are critical for salt resistance in plants, an important issue in view of the spread of arid soils^7^.

**Figure 1:**
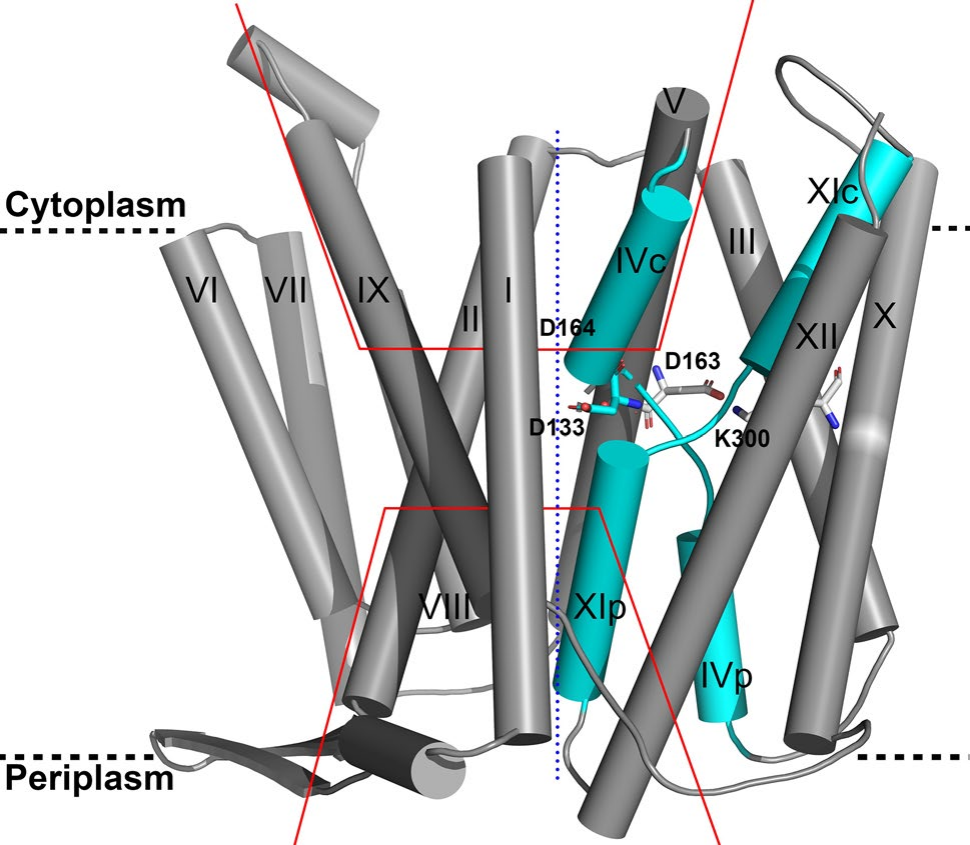
The putative Na^+^ binding site of NhaA. The crystal structure of NhaA (PDB entry 4ATV) is viewed parallel to the membrane as cylinder representation. The TMs are numbered by Roman numerals and packed into two domains (interface, left side and core, right side). Their separation is indicated by the blue dotted line. The NhaA fold, with topologically inverted TM IV and TM XI colored cyan is crossing each other where they are interrupted by an unwound portion (extended chains) that split them into cytoplasmic (c) and periplasmic (p) segments. The other TMs are colored grey. The cytoplamsic and periplasmic funnels are indicated by red lines, and the membrane by broken black lines. Functional important residues (Asp133, Asp163, Asp164, and Lys300) at the proposed binding site are shown in stick representation.

NhaA, the Na^+^/H^+^ antiporter of *Escherichia coli* is the main antiporter responsible for homeostasis of the Na^+^ and H^+^ concentrations in the bacterial cell^2^. NhaA homologues exist in many bacteria^8^, including enterobacterial pathogens^9,10^. NhaA is most extensively studied, and its crystal structure has been determined^11^, followed by the structural determination of its homo- and heterologues^12,13^. NhaA is a homodimer^12,14–16^, and its momomeric structure^11^ (Fig. 1) has provided key structural insights into the function and regulation of this class of antiporters. It opened the way to structure-based interdisciplinary studies that otherwise could not have been carried out. Monomeric NhaA is comprised of 12 transmembrane helices (TMs)^11^ in unique fold (termed the NhaA fold)^17^ that are packed in two domains: the interface domain, which connects the two monomers of NhaA in a dimer (TMs I, II, VI–IX), and the core domain, which is involved in ion translocation (TMs III–V and X–XII). Despite very low homology between TMs III–V and TMS X–XII, they represent topologically inverted repeats. Between the interface and core domains there are two cavities (termed the funnels) facing the cytoplasm and periplasm on either side of the protein (Fig. 1). Furthermore, extended chains, that cross each other in the middle of the membrane, interrupt TMs IV and XI (the so-called TM IV/XI assembly) each into small helices facing either the cytoplasm (TM IVc and XIc) or periplasm (TMIVp and XIp) (Fig. 1). Because the extended chains are not completely hydrogen-bonded, they can participate in ion binding and create a very delicately balanced electrostatic environment in the middle of the membrane, crucial for NhaA’s mechanism of action^11^. The number of secondary transporters, including non-homologs of NhaA, that share the NhaA fold is steadily increasing^13,17^.

NhaA is a "nano machine" that exchanges one Na^+^ (or its surrogate Li^+^) for 2 H^+^ across the membrane^18–20^. It has a very rapid turnover rate and is drastically sensitive to pH^18^, a property it shares with both prokaryotic and eukaryotic Na^+^/H^+^ antiporters^8^. As a secondary transporter, NhaA harnesses the energy of the H^+^ electrochemical gradient, across the membrane, by allowing downhill movement of H^+^ to drive the uphill antiport of Na^+^ (or Li^+^) against their electrochemical gradient^19,20^.

NhaA’s transport mechanism is believed to follow the conceptual alternating access model^21^, according to which conformational changes of the transporter expose the active site to either side of the membrane in alternating fashion. Hence, to understand the antiport mechanism, it is critical to gain insight into the individual reaction steps that comprise the transport cycle and their dynamics, the identification of the binding site(s) for Na^+^ and Li^+^, and how cation binding is regulated and connected to other steps in the antiport cycle. However, to date, crystallographic support for the precise location of the cation binding site is still missing^11,12^. Nevertheless, many indirect observations have predicted that the Na^+^/Li^+^ binding site includes Asp163 and Asp164 of TM V (Fig. 1). These residues are highly conserved as recently further analyzed by evolutionary analysis of the flood of 6597 putative antiporters sequences^22^. Furthermore, mutagenesis studies revealed that both residues are functionally irreplaceable^23–26^. Asp164 is exposed to the cytoplasm via the cytoplasmic funnel^11^, and both aspartates are located near the crossing between the extended chains of the TM IV/XI assembly. In ASBT, a homologue that shares the NhaA fold, the Na^+^ binding site was identified in this region^27^. Whereas Li^+^ binding to affinity-purified NhaA in detergent micelles using isothermal calorimetry (ITC) combined with mutagenesis^20,28^ revealed, consistent with established uptake studies, strong pH dependence of Li^+^ binding to NhaA, this approach failed to provide data for Na^+^ binding. The failure to obtain ITC data for Na^+^ binding most likely stems from two main reasons: (i) the Na^+^ binding affinity appears to be an order of magnitude lower than that for Li^+^ as deduced from the apparent *K*_m_ of the ions in transport assays (both in membrane vesicles and purified NhaA reconstituted in proteoliposomes, the apparent *K*_m_ for Na^+^ transport is 0.2–0.5 mM and that of Li^+^ is 0.02 mM^29^), and (ii) NhaA is a functional dimer; whereas the ITC measurements were performed in the presence of *n*-dodecyl-β-D-maltopyranoside (DDM), recent studies revealed that DDM does not support NhaA dimerization that is required for optimal activity and stability^30^. Specifically increasing the concentration of DDM above 0.1% (w/w) delipidates NhaA at the dimer interface and splits the NhaA dimers into monomers while addition of cardiolipin reconstitutes the dimers. Also, using mass spectroscopy showed that cardiolipin binds to the NhaA dimer interface^31^, thereby stabilizing the NhaA dimer^30^.

Notwithstanding the importance of the NhaA Li^+^ binding results, Na^+^, rather than Li^+^, is the most prevalent cation in nature, and excreting Na^+^ from the cytoplasm is the main physiological role of NhaA. NhaA maintains a cytoplasmic Na^+^ concentration of around 0.5 mM^32^, a value which is consistent with the apparent *K*_m_ for Na^+^ transport^29^. However, an apparent Na^+^ binding affinity of 1–10 mM was previously estimated using solid supported membranes (SSM)-based electrophysiological measurements and competition between Na^+^ and H^+^ at the binding site has been suggested to underpin the pH regulation of NhaA^33,34^, excluding the previously suggested option of a pH sensor^35^. Hence, there is no consensus regarding the precise location, organization and the determinants of the Na^+^ (and Li^+^) binding site of NhaA, and whether the pH controls Na^+^ binding and/or transport at physiological cytoplasmic Na^+^ concentrations by controlling a rate-limiting step in the Na^+^ transport cycle.

To address these questions, here we applied the scintillation proximity-based radioligand-binding assay (SPA) to follow direct ^22^Na^+^ binding^36^ to affinity-purified NhaA. This assay was previously successfully used for the determination of the Na^+^ binding kinetics of many Na^+^-dependent transport proteins^37–41^. Our results provide insight into the molecular determinants of the long-sought direct interaction of Na^+^ with NhaA. These data have wide ramifications for studies focusing on Na^+^ resistance in plants or the development of drugs that target Na^+^/H^+^ antiporters in humans.

## Results and discussion

### Assay optimization

**Figure 2:**
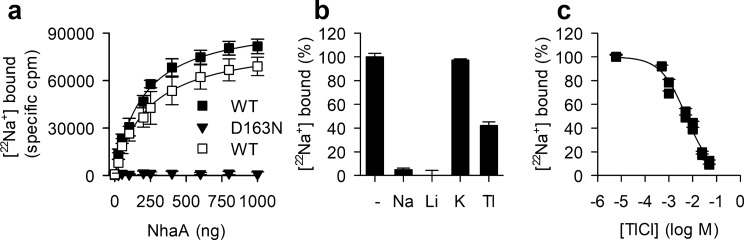
Ion binding by NhaA. (**a**) Optimization of the scintillation proximity assay (SPA) for ^22^Na^+^ binding by NhaA. PVT His-tag SPA beads were resuspended in the SPA assay buffer (200 mM Tris-Mes pH 8.0, 20% glycerol, 0.1 mM TCEP plus the indicated concentrations of DDM and/or cardiolipin) at a final concentration of 1.25 mg/mL. Binding of 50 µM [^22^Na]Cl (50 Ci mol^−1^) was performed for 30 min with the indicated amounts of affinity-purified His-tagged NhaA at 23 °C before determination of the counts-per-minute (cpm) in the SPA mode of the MicroBeta 1450 microplate reader. The non-proximity (background) signal was determined by performing the reaction in the presence of 800 mM imidazole which competes with the His-tagged protein for binding to the His-tag SPA beads. Binding was tested for NhaA-WT in assay buffer that contained either 0.1% (w/v) DDM (open square) or 0.015% (w/v) DDM and 5% (w/v) cardiolipin (filled square). Binding was also tested for NhaA-D163N in the presence of 0.015% (w/v) DDM and 5% (w/v) cardiolipin (filled inverted triangle). Data of three independent experiments (represented as mean ± s.e.m.) were subjected to global nonlinear regression fitting in Prism 8 to obtain the total binding values (*B*_*max*_) (85,570 ± 2,528 specific cpm for NhaA-WT in 0.1% DDM and 98,980 ± 2,992 specific cpm for NhaA-WT in 0.015% DDM and 5% cardiolipin, respectively) and *EA*_50_ (effective amount of NhaA at 50% *B*_*max*_) of 236.2 ± 19.71 ng and 193.9 ± 17.87 ng, respectively. All subsequent experiments were performed in the presence of 200 ng of NhaA, the protein amount that occupies about 50% of the SPA beads in the assay to prevent depletion of the radioligand or achieve full occupancy of the SPA beads. (**b**) Interaction of NhaA with different cations. SPA-based equilibrium binding of 50 µM [^22^Na]Cl (50 Ci mol^−1^) to 200 ng of NhaA-WT was performed in the presence or absence of 10 mM of NaCl (Na), LiCl (Li), KCl (K), or TlCl (Tl). The assay was performed in assay buffer containing 0.015% (w/v) DDM and 5% cardiolipin and normalized to the specific cpm measured in the absence of ionic additions (−). (**c**) Effect of increasing concentrations of the halide Tl^+^ (chloride salt) on the binding of 50 µM [^22^Na]Cl to 200 ng of NhaA-WT. Data of three independent experiments are shown as mean ± s.e.m. in panels b and c. Data in panel c were subjected to global nonlinear regression fitting in Prism 8, yielding an ${IC}_{50}^{{Tl}^{+}}$ of 10.97 mM with a Hill coefficient of − 0.61. See Table 1 for the results in log and non-log scale.

We chose to determine the binding properties of ^22^Na^+^ to affinity-purified NhaA in DDM micelles using the SPA technique, which allows for the rapid and sensitive measurement of radioisotope binding without the need for a separation step^36^. For this purpose, we first optimized the amount of affinity-purified NhaA in the presence of 0.1% (w/v) DDM for the binding assay (Fig. 2a). Since it was shown that high concentrations of DDM delipidate NhaA, leading to the separation of the NhaA dimer into the monomeric form and reduced Na^+^/H^+^ antiport activity, while cardiolipin restores dimerization in vivo and *in vitro*^30^, we performed this assay in the presence of 0.015% DDM and 5% cardiolipin (Fig. 2a). Whereas half-maximum saturation of 125 µg of the His-tag polyvinyl toluene (PVT) SPA beads in the assay format was reached between 150 and 200 ng of NhaA, a value that was independent of the experimental condition, maximum binding in 0.015% DDM/5% cardiolipin was about 10% higher than that observed when the assay was performed in the presence of 0.1% DDM. Testing the effect of the protein amount used in the binding assay for NhaA-D163N, a mutant of NhaA that features impaired Na^+^/H^+^ antiport activity^42^ revealed that only negligible binding of 50 µM ^22^Na^+^ above the non-proximity signal was observed, even when 1 µg of the mutant protein was used in the assay (Fig. 2a). The results of these pilot experiments documented that the SPA is a suitable platform to determine the direct binding of Na^+^ to NhaA, an approach that was hitherto not possible with other methods.

### Ion recognition by NhaA

Under physiological conditions NhaA specifically exports 1 Na^+^ or 1 Li^+^ from the cell in exchange for the cellular import of 2 H^+^^19,20^, and, thus, is electrogenic as in each reaction cycle one net positive charge enters the cell. As mentioned above, the apparent antiport *K*_m_ for Na^+^ is ~ 0.5 mM, whereas that of Li^+^ is ten-fold lower (~ 0.02 mM) in isolated everted membrane vesicles and NhaA proteoliposomes^2^.

Building on the feasibility of the SPA to measure equilibrium binding of ^22^Na^+^ with NhaA, we next performed measurements to determine the interaction of different ions with NhaA. To test whether non-radioactive ions compete for binding of radiolabeled Na^+^ with NhaA, we performed a competion assay in which NaCl, LiCl, KCl, and TlCl were added to the assay, and the reduction of ^22^Na^+^ binding was monitored. Previous transport measurements suggest that Na^+^ and Li^+^ bind to the same site in NhaA^19^. In contrast, there is no evidence that supports the interaction of K^+^ with NhaA. However, although the ion radius of the monovalent halide Tl^+^ (1.5 Å) is similar to that of K^+^ (1.44 Å) but significantly larger than that of Na^+^ (1.12 Å)^43,44^, Tl^+^ was identified in the Na^+^ binding sites of the Na^+^/H^+^ antiporter of *Pyrococcus abyssi* (PaNhaP)^45^ and in the Na^+^-dependent aspartate transporter Glt_Ph_^46^. Thus, it seemed feasible to speculate that Tl^+^ can also interact with NhaA of *E. coli*.

Binding of 50 µM [^22^Na]Cl was measured in the presence of non-labeled NaCl, LiCl, KCl, and TlCl at 10 mM, a concentration that is about 20- and 500-fold larger than the reported apparent affinities for the antiport reaction of Na^+^ and Li^+^, respectively. Based on the results of the pilot experiments (Fig. 2a), and to maintain a membrane-mimicking environment that was used for the cell (or proteoliosome)-based uptake studies^19,20^, we performed the assay in the presence of 0.015% DDM and 5% cardiolipin. Figure 2b shows that whereas binding of 50 µM [^22^Na]Cl in the presence or absence of 10 mM KCl exhibited virtually identical levels, 10 mM NaCl or LiCl reduced binding by 95.1% and 99.8%, respectively. Binding of 50 µM [^22^Na]Cl was reduced to 42.1% when the assay was performed in the presence of 10 mM TlCl, revealing that Tl^+^ can indeed compete with Na^+^ for binding to NhaA. To further investigate the binding kinetics of Tl^+^ we determined the apparent affinity of Tl^+^ binding to NhaA (expressed as the ${IC}_{50}^{{Tl}^{+}}$, the concentration of Tl^+^ that yields 50% reduction of ^22^Na^+^ binding) by measuring the effect of increasing concentrations of Tl^+^ on 50 µM [^22^Na]Cl binding. Fitting the isotherm of ^22^Na^+^ displacement by increasing concentrations of Tl^+^ (Fig. 2c) yielded an ${IC}_{50}^{{Tl}^{+}}$ of ~ 11 mM (see Table 1 for the results of the nonlinear regression fitting in log and non-log scale). Furthermore, the calculated Hill coefficient of ~ 0.61, i.e., below unity, indicates the lack of cooperativity for Tl^+^ binding, suggesting the presence of only one Tl^+^ binding site. Given the complexity associated with the determination of the accurate Hill coefficient, it must be noted that this coefficient reflects cooperativity rather than the absolute number of binding sites in proteins^37–41^.

**Table 1:** Summary of nonlinear regression fitting to obtain the (apparent) affinities for Tl^+^, Na^+^, or Li^+^ binding.

Condition	Log ${IC}_{50}^{{Tl}^{+}}$ (M)	Hill slope	${IC}_{50}^{{Tl}^{+}}$(mM)	*R*^2^	Range
**Tl**^**+**^					
0.015 DDM/5% cardiolipin	− 1.959929699 ± 0.273905651	− 0.61 ± 0.13	10.97	0.9864	− 20.275–101.6
**Na**^**+**^					
0.1 DDM	− 2.671786305 ± 0.063454972	− 0.72 ± 0.06	2.13	0.9880	− 3.366–84.18
0.015 DDM	− 3.234476345 ± 0.021847845	− 1.14 ± 0.06	0.58	0.9956	2.043–90.18
0.1 DDM/5% cardiolipin	− 3.354067538 ± 0.02400254	− 1.11 ± 0.07	0.44	0.9945	0.5002–95.25
0.015 DDM/5% cardiolipin	− 3.369373143 ± 0.017169354	− 1.21 ± 0.06	0.42	0.9968	2.495–100.4
**Li**^**+**^					
0.1 DDM	− 3.293734146 ± 0.047545777	− 0.95 ± 0.10	0.51	0.9838	3.489–82.48
0.015 DDM	− 3.511544709 ± 0.038462626	− 0.85 ± 0.07	0.31	0.9898	− 1.531–91.91
0.1 DDM/5% cardiolipin	− 3.627689929 ± 0.028581723	− 0.83 ± 0.05	0.24	0.9945	− 0.7337–98.34
0.015 DDM/5% cardiolipin	− 3.76810917 ± 0.04001757	− 0.99 ± 0.09	0.17	0.9858	0.5753–99.97

**Figure 3:**
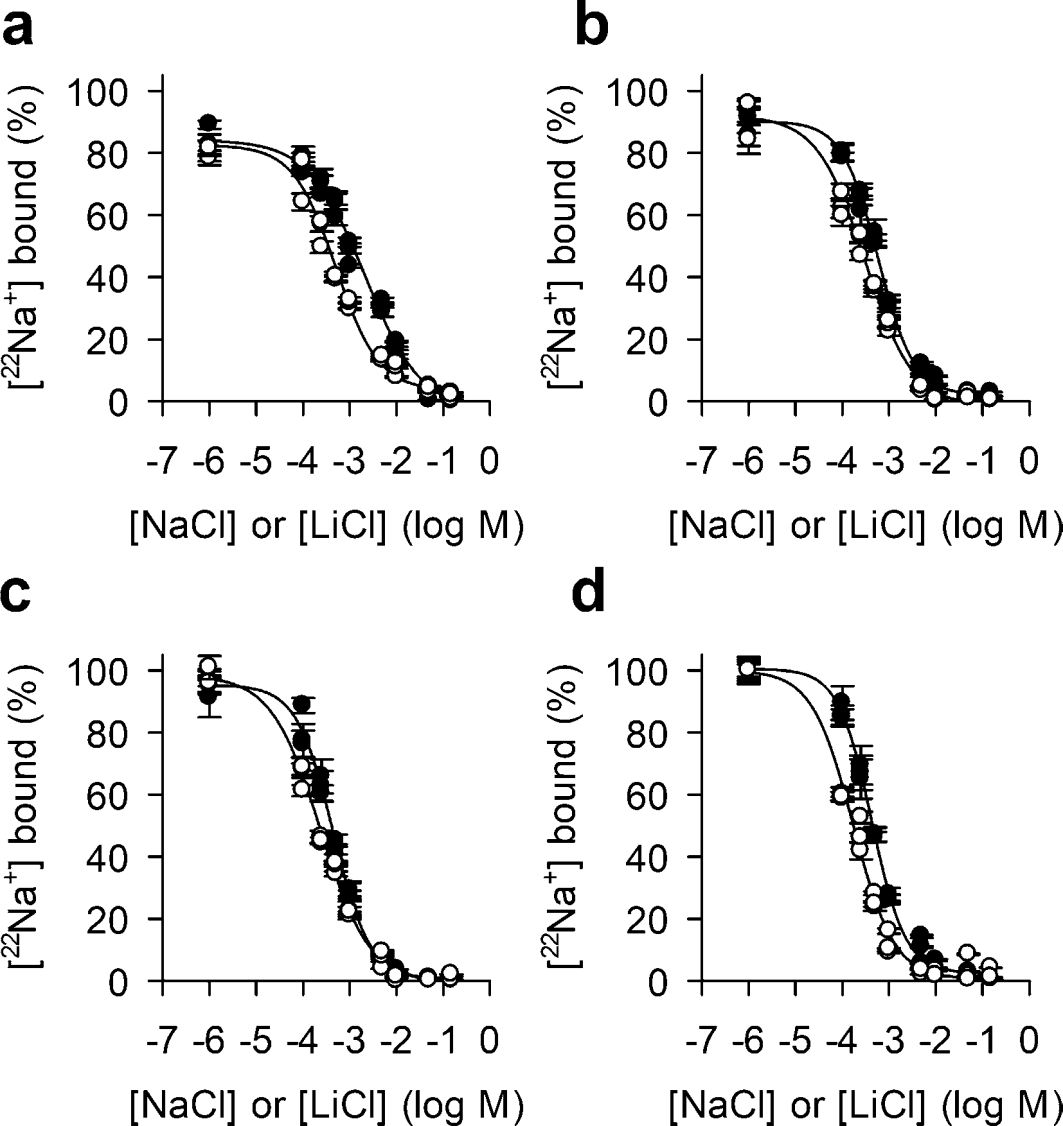
The dependence of the apparent binding affinities of Na^+^ and Li^+^ on the experimental conditions. The apparent binding affinities of Na^+^ (solid symbols) and Li^+^ (open symbols) were determined by isotopic displacement of 50 µM [^22^Na]Cl (50 Ci mol^−1^) bound to 200 ng NhaA-WT with increasing concentrations of NaCl (yielding the ${EC}_{50}^{{Na}^{+}}$) or by displacing bound 50 µM [^22^Na]Cl (50 Ci mol^−1^) with increasing concentrations of LiCl (yielding the ${IC}_{50}^{{Li}^{+}}$). The assay was performed in buffer containing (**a**) 0.1% (w/v) DDM, (**b**) 0.015% (w/v) DDM, (**c**) 0.1% (w/v) DDM plus 5% (w/v) cardiolipin, or (**d**) 0.015% (w/v) DDM plus 5% (w/v) cardiolipin. Data of three independent experiments (presented as mean ± s.e.m.) were subjected to global nonregression fitting and the data of the fits are shown in Table 1.

To compare the apparent affinity of Na^+^ and Li^+^, the two only cations that were previously shown to be exchanged with H^+^ by NhaA^19,20^, we performed assays in which increasing concentrations of NaCl or LiCl were used to displace 50 µM [^22^Na]Cl bound to NhaA. Like for Tl^+^, we determined the ${IC}_{50}^{{Li}^{+}}$ as measure for the apparent affinity of Li^+^, whereas we determined the ${EC}_{50}^{{Na}^{+}}$, representing the effective concentration of NaCl to isotopically replace 50% of bound ^22^Na^+^. Taking into account recent findings that highlight the importance of the lipidation state of NhaA^30^, and the results from the pilot experiments (Fig. 2a), implying that the binding activity of NhaA is correlated to the assay conditions, we determined these measurements in the presence of two different concentrations of DDM (0.1% or 0.015%) and in the presence or absence of 5% cardiolipin. Binding of 50 µM [^22^Na]Cl to 200 ng NhaA increased in the following order: 0.1% DDM (Fig. 3a) < 0.015% DDM (Fig. 3b) < 0.1% DDM and 5% cardiolipin (Fig. 3c) < 0.015% DDM and 5% cardiolipin (Fig. 3d); likewise, the ${EC}_{50}^{{Na}^{+}}$ and the ${IC}_{50}^{{Li}^{+}}$ decreased in the same order, meaning that the highest affinity for Na^+^ and Li^+^ was observed in 0.015% DDM plus 5% cardiolipin (Table 1). Also, the Hill coefficients of the fits for 50 µM [^22^Na]Cl replacement by Na^+^ or Li^+^ were close to unity, reflecting the absence of binding cooperativity as would be expected for a single cation binding site.

Importantly, the ${EC}_{50}^{{Na}^{+}}$ for Na^+^ was found to be ~ 0.4 mM when the assay was performed with a low DDM concentration (0.015%) and in the presence of cardiolipin, similar to the apparent *K*_*m*_ for Na^+^ transport determined in proteoliposomes and membrane vesicles. In contrast, when the assay was performed in the presence of a higher concentration (0.1%) of DDM and in the absence of cardiolipin, the ${EC}_{50}^{{Na}^{+}}$ increased about five-fold (~ 2 mM). Likewise, a similar trend was observed when LiCl was used to displace bound 50 µM [^22^Na]Cl to determine the ${IC}_{50}^{{Li}^{+}}$, yielding values of ~ 0.5 mM or ~ 0.17 mM in 0.1% DDM or 0.015% DDM/5% cardiolipin, respectively. Hill coefficients of ~ 1 suggest the presence of one Li^+^ binding site (Table 1). These data point out that the higher binding activity observed in the pilot experiments when the assay was performed in 0.015% DDM/5% cardiolipin (Fig. 2a) cannot be attributed to the specific effect of these two compounds on Na^+^ binding per se in the assay, but they rather reflect that lipids play a critical role in the functionality of NhaA, consistent with a recent study^30^. Remarkably, the apparent affinity found here for Na^+^ (~ 0.4 mM) equals the physiological intracellular Na^+^ concentration^32^, supporting the contention that NhaA is the main antiporter in Na^+^ homeostasis of *E. coli* and possibly similar bacterial cells. On the other hand, for unknown reason, the apparent affinity of Li^+^ (0.17 mM) found here is ten-fold lower than expected from the apparent *K*_m_ for Li^+^ transport in isolated membranes or NhaA proteoliposomes (0.02 mM).

### Effect of pH on Na^+^ binding by NhaA

**Figure 4:**
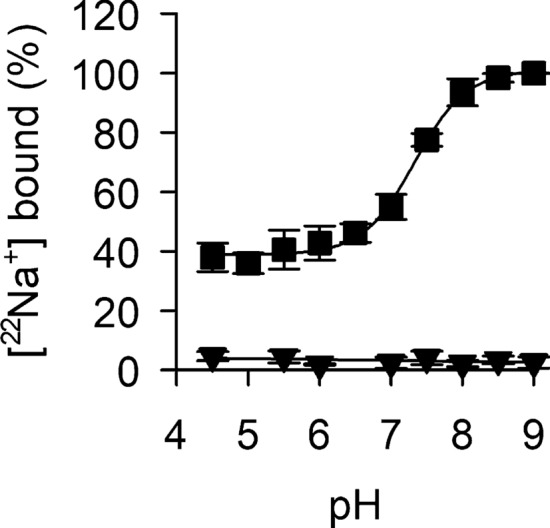
pH dependence of Na^+^ binding to NhaA. Binding of 50 µM [^22^Na]Cl (50 Ci mol^−1^) to 200 ng NhaA-WT (filled square) or NhaA-D163N (filled inverted triangle) was measured in assay buffer (containing 0.015% (w/v) DDM plus 5% (w/v) cardiolipin) with the indicated pH. Data of three independent experiments are shown as mean ± s.e.m. and plotted as a function of pH.

Since it was shown that the antiport acitivity of NhaA is drastically dependent on the pH^18^, this dependence may reflect the pH-mediated regulation of either the passage of Na^+^ through NhaA during the transport process across the membrane or the binding of Na^+^ to NhaA or both. All options seem feasible based on described mechanisms of ion-coupled transport^13^ that invloves the exchange of two H^+^ with one Na^+^ (or Li^+^)^19,20^. Since it has been suggested that the Na^+^ (or Li^+^) binding site also binds H^+^^34^, a high H^+^ concentration (low pH) may block access of Na^+^ (or Li^+^) to such a site that is shared for binding with H^+^, thereby reflecting an apparent pH regulation through competetion for the same site by the different ions. Consistent with this notion, previous ITC measurements^20,28^ showed that Li^+^ binding occurs only at pH values ≥ 8.0. However, antiport data showing significant H^+^/Na^+^ (or Li^+^) antiport activity by NhaA wild-type at pH ≥ 6.5, thus suggesting that differences in the experimental conditions between the different assays may (partially) obscure the pH dependence of NhaA. In particular, the ITC experiments were performed at higher detergent concentration and in the absence of cardiolipin, conditions that were shown to interfere with the functionally important dimerization of NhaA^30^. Whereas such a competetive mechanism between H^+^ and Na^+^ (or Li^+^) binding for the same site may exist, it cannot be the only scenario of pH regulation as our SPA analysis shows that about 50% of Na^+^ binds in a pH independent fashion (Fig. 4). Our SPA study reports equilibrium binding of ^22^Na^+^ to detergent-solubilized NhaA under non-compartmentalized conditions; it displays the interaction of Na^+^ with NhaA in the absence of any vectorial transmembrane ion gradient (i.e., ΔpH, $\Delta {\stackrel{\sim }{\mu }}_{{H}^{+}}$) that may control the state of the protein in Na^+^-binding or non-binding conformation but rather reflects the protonation state of the cation active site at the experimentally selected pH. The results summarized in Fig. 4 show that: (i) direct Na^+^ binding to NhaA is drastically dependent on the pH, consistent with the results of the antiport measurements^18^. Maximum Na^+^ binding was observed when the H^+^ concentration was low (pH ≥ 8.0), indicating a tight regulation of Na^+^ binding to NhaA by H^+^; (ii) between pH 6.0 and 7.0 the binding is about 50% of that observed at pH values ≥ 8.0, with a progressive increase in binding between pH 7.0 and pH 8.0. However, given the fact that ^22^[Na^+^] binding of about 40% was observed at high H^+^ concentration (pH ≤ 6.0) when compared to that measured at pH ≥ 8.0, this result points out that fractional Na^+^ binding to NhaA can occur in pH-independent manner. This finding contradicts a mechanism in which Na^+^ binding is exclusively controlled by the deprotonation of residues in Na^+^ coordination, i.e., by ion competetion^12^; (iii) testing ^22^Na^+^ binding by NhaA-D163N at different pH values (Fig. 4) resulted in ^22^Na^+^ binding signals that were virtually indistinguishable from those obtained in the presence of imidazole, the agent that competes with the His-tagged NhaA for binding to the PVT His-tag SPA beads (Fig. 2a). This result revealed this mutant’s inability to bind Na^+^ at any pH tested and implies that the lack of Na^+^/H^+^ antiport activity^42^ can be attributed to this NhaA mutant’s inability to bind Na^+^, thus identifying Asp163 as direct Na^+^ coordinating partner. Since fractional Na^+^ binding by NhaA wild-type was observed at low pH, fractional Na^+^ binding would be expected for NhaA-D163N if the mutation would impact H^+^ binding, thus ruling out an effect of the mutation on H^+^ binding that, in turn, would impair Na^+^ binding or transport. Taken together, these results raise the question as to which residues are involved in pH dependent and pH independent Na^+^ binding.

Despite the fact that to date a high-resolution structure of Na^+^ (or Li^+^)-bound NhaA is missing, biochemical and biophysical measurements, as well as molecular dynamics (MD) simulations indicate that Asp163 and Asp164 are involved in Na^+^ binding^12,28^. Since Asp164 is strictly conserved in all Na^+^/H^+^ antiporters (including those that mediate the electroneutral stoichiometric exchange of 1 Na^+^ and 1 H^+^), this residue is generally accepted, in addition to being a Na^+^ binding site, as the conserved H^+^ binding site in this class of proteins^22,28,34,47^. Previous studies suggest that the second H^+^ (in the electrogenic Na^+^/H^+^ antiporters like NhaA) may bind to Asp133 or Asp163^48^. However, Asp133 is despensable for function^25,28^ and has been shown to contribute its peptide-bond carbonyl to bind Na^+^^49^. Furthermore, state-of-the-art continuous constant pH molecular dynamics simulations of NhaA K300 mutants suggested that in these mutants the electrogenic transport is maintained by utilizing Asp133 as an alternative proton-binding residue^50^. Therefore, and based on the recent evolutionary anlysis and metadynamics simulations^51^, it seemed feasible to speculate that the irreplaceable Asp163 (like Asp164) possesses a central dual function as binding partner for H^+^ and Na^+^ of the electrogenic (2 H^+^/1 Na^+^) exchange mechanism^52^. The crystal structure of the dimeric NhaA and MD simulations point out the formation of a salt bridge between Asp163 and Lys300 in NhaA that is interrupted when Asp163 participates, together with Asp164, in the binding of Na^+^^12^. In this vein, Lys300 was shown to be important in pH regulation and antiport activity of NhaA^26^ mainly because the salt bridge is important for NhaA stability^53^. In fact, in the NhaA homolog Na^+^/H^+^ antiporter NhaP from *Pyrococcus abyssi* (PaNhaP), which was crystallized in ion-bound state (here, the Na^+^ surrogate Tl^+^ was used), the ion binding site was characterized as distorted trigonal bpyramide^45^. Here, ion coordination is achieved through the direct interaction of the carboxyl groups of Glu73 and Asp159, whereas the ion interacts through a bound water molecule with Asp130 and with the hydroxyl side chain of Ser155 and the main-chain carbonyl of Thr129. A similar trigonal bpyramidal Na^+^ coordination was identified for the ion in the Na2 site in LeuT, whereas Na^+^ in the Na1 site of LeuT features an octahedral coordiantion^54^, pointing to the involvement of ≥ six coordination partners for Na^+^. As our data shows that Tl^+^ binds to NhaA (Fig. 2), it seems interesting to propose that Na^+^ (or Li^+^) coordination in NhaA, like that in PaNhaP^45^ or LeuT^54^, involves additional interactions with amino acid side chains and main chains that cannot be protonated/deprotonated. Consequently, such a coordination network for Na^+^ (or Li^+^) that is insensitive to H^+^ may serve as binding scaffold for Na^+^ (Li^+^) in pH independent manner. In such a scenario it appears possible that deprotonation of protonable side chains of residues that are involved in Na^+^ binding provide the activation energy that is required for the execution of the full H^+^/Na^+^ exchange cycle.

In summary, our SPA-based direct Na^+^ binding to NhaA data revealed that (i) the Na^+^ binding affinity of NhaA is ~ 0.4 mM, implying that NhaA is the main antiporter for Na^+^ homeostasis at 0.4 mM, the cytoplasmic concentration of *E. coli* and possibly similar cells, (ii) the membrane lipid cardiolipin plays a critical role in the cation binding functionality of NhaA, (iii) the halide Tl^+^ interacts with NhaA, (iv) whereas acidic pH inhibits maximum binding of Na^+^ to NhaA, partial Na^+^ binding by NhaA is independent of the pH, an important novel insight into the effect of pH on NhaA cation binding.

By gaining insight into the molecular determinants of the interaction of Na^+^ with NhaA, these findings provide valuable information for the generation of more accurate mechanistic models of an essential reaction that is maintained throughout organisms from bacteria to man. Thus, these data have wide ramifications for studies focusing on Na^+^ resistance in plants or the development of drugs that target Na^+^/H^+^ antiporters in humans, e.g., in the treatment of hypertension and congestive heart failure.

## Experimental procedures

### Plasmids, bacterial strains, and culture conditions

pAXH3^55^ is a plasmid expressing His-tagged NhaA. TA16 is an *E. coli* K-12 derivative (*nha*A^+^, *nha*B^+^, *lac*I^Q^, Δ*lac*ZY, *thr*1^56^) and is otherwise isogenic to EP432 (*nha*A^-^, *nha*B^-^, *lac*I^Q^, Δ*lac*ZY, *thr*1)^18,55^. Cells were grown in modified L broth (LBK)^57^. For plates, 1.5% agar was used. For overexpression, the cells were grown in minimal medium A^57^ without sodium citrate and with 0.5% (w/v) glycerol, 0.01% (w/v) MgSO_4_·7H_2_O, and 2.5 μg/mL thiamine. Antibiotic was 100 μg/mL ampicillin. Cells were grown to A_600_ 0.6, induced with 0.5 mM isopropyl-1-thio-β-D-galactopyranoside, grown for 2 h to A_600_ 1–1.2, harvested, and used for preparation of high pressure membranes^57^ either after storage overnight at 4 °C or after freezing at − 20 °C.

### Overexpression and affinity purification of NhaA protein

Overexpression of NhaA^58^, isolation of high-pressure membrane vesicles^57^, and immobilized metal-affinity purification (Ni^2+^-nitrilotriacetic acid-agarose, Qiagen)^59^ were performed as described previously, but the protein was eluted in a buffer (pH 7.9) containing 10% glycerol, 300 mM imidazole, 25 mM citric acid, 100 mM choline chloride, 5 mM MgCl_2_, and 0.015% DDM. Sucrose (10%) was added to the eluted protein solution, and the solution was dialyzed overnight at 4 °C in acidic dialysis buffer containing 25 mM potassium citrate (pH 4.0), 10% sucrose, 100 mM choline chloride, 25 mM citric acid, 5 mM MgCl_2_, 0.015% DDM and was frozen at – 80 °C.

### Detection and quantification of NhaA

Total quantities of membrane- and affinity-purified proteins were determined using the Bradford assay^60^. In certain cases, quantitation of the affinity-purified NhaA was carried out by resolving the sample and a sample of known NhaA concentration on SDS-PAGE, staining the gels with Coomassie Blue and quantifying the band densities using Image Gauge (Fuji) software.

### SPA-based binding assay

Binding of radiolabeled Na^+^ to His-tagged NhaA was measured with the SPA method^36^. The assay was modified to test the effect of detergent (DDM) and cardiolipin (CL) on NhaA’s binding activity. Basic assay conditions, unless otherwise indicated, involved the immobilization of 200 ng of affinity-purified NhaA to Cu^2+^-coated polyvinyl toluene (PVT) SPA beads (at a concentration of 1.25 mg ml^−1^) in 100 µL assay buffer composed of 200 mM tris(hydroxymethyl)aminomethane (Tris)/2-(N-morpholino)ethanesulfonic acid (Mes), pH 8.0, 20% glycerol, 0.1 mM tris(2-carboxyethyl)phosphine (TCEP; Sigma), 0.1% (w/v) DDM (Anatrace). When indicated, the concentration of DDM was reduced to 0.015% (w/v) and/or cardiolipin was added at a concentration of 5% (w/v). Optimized binding conditions were evaluated by varying the amount of NhaA from 25 to 1000 ng per 100-µL assay (Fig. 2a). Unless otherwise indicated, binding of 50 µM [^22^Na]Cl (21.03 Ci mmol^−1^; Perkin Elmer) was assayed in individual wells of clear-bottom/white-wall 96-well plates. Binding was performed in the dark at 23 °C with vigorous shaking on a vibrating platform. Varying the incubation periods revealed that [^22^Na]Cl binding reached equilibrium within 1 min, and the binding activity remained constant for a minimum of 24 h. The counts per minute (cpm) were determined in the SPA mode of a Wallac 1450 MicroBeta photomultiplier tube (PMT) plate counter. The non-proximity background signal was determined for each sample in the presence of 800 mM imidazole, which prevents the interaction of the His-tagged protein with the Cu^2+^-coated SPA beads, and the non-proximity cpm was subtracted from the cpm determined in the absence of imidazole to obtain the specific binding activity. Data were normalized with regard to the activity of wild-type NhaA in the absence of non-labelled ligand and was set as 100%. All experiments were performed at least in triplicate with technical replicas of 3. Data were expressed as mean ± s.e.m. Data fits of binding isotherms were performed using unweighted, unconstrained nonlinear regression algorithms in Prism 8 (GraphPad), and errors represent the s.e.m. of the fit. The goodness of the fit (*R*^2^) and the range are shown in Table 1.

## References

[1] Orlowski J, Grinstein S (2007). Emerging roles of alkali cation/proton exchangers in organellar homeostasis. Curr. Opin. Cell Biol.

[2] Padan E (1837). Functional and structural dynamics of NhaA, a prototype for Na^+^ and H^+^ antiporters, which are responsible for Na^+^ and H^+^ homeostasis in cells. Biochim. Biophys. Acta.

[3] Padan E, Landau M (2016). Sodium-proton (Na^+^/H^+^) antiporters: properties and roles in health and disease. Met. Ions Life Sci..

[4] Kondapalli KC, Kallay LM, Muszelik M, Rao R (2012). Unconventional chemiosmotic coupling of NHA2, a mammalian Na^+^/H^+^ antiporter, to a plasma membrane H^+^ gradient. J. Biol. Chem..

[5] Deisl C (2013). Sodium/hydrogen exchanger NHA2 is critical for insulin secretion in beta-cells. Proc. Natl. Acad. Sci. USA.

[6] Fuster DG, Alexander RT (2014). Traditional and emerging roles for the SLC9 Na^+^/H^+^ exchangers. Pflugers Arch..

[7] Bassil E, Blumwald E (2014). The ins and outs of intracellular ion homeostasis: NHX-type cation/H^+^ transporters. Curr. Opin. Plant Biol..

[8] Krulwich TA, Sachs G, Padan E (2011). Molecular aspects of bacterial pH sensing and homeostasis. Nat. Rev. Microbiol..

[9] Lescat M (2014). The conserved *nhaAR* operon is drastically divergent between B2 and non-B2 *Escherichia coli* and is involved in extra-intestinal virulence. PLoS ONE.

[10] Minato Y (2013). Na^+^/H^+^ antiport is essential for *Yersinia pestis* virulence. Infect. Immun..

[11] Hunte C (2005). Structure of a Na^+^/H^+^ antiporter and insights into mechanism of action and regulation by pH. Nature.

[12] Lee C (2014). Crystal structure of the sodium-proton antiporter NhaA dimer and new mechanistic insights. J. Gen. Physiol..

[13] Shi Y (2013). Common folds and transport mechanisms of secondary active transporters. Annu. Rev. Biophys..

[14] Gerchman Y, Rimon A, Venturi M, Padan E (2001). Oligomerization of NhaA, the Na^+^/H^+^ antiporter of *Escherichia coli* in the membrane and its functional and structural consequences. Biochemistry.

[15] Hilger D, Polyhach Y, Padan E, Jung H, Jeschke G (2007). High-resolution structure of a Na^+^/H^+^ antiporter dimer obtained by pulsed electron paramagnetic resonance distance measurements. Biophys. J..

[16] Williams KA, Geldmacher-Kaufer U, Padan E, Schuldiner S, Kuhlbrandt W (1999). Projection structure of NhaA, a secondary transporter from *Escherichia coli*, at 4.0 Å resolution. Embo J..

[17] Padan E (2015). NhaA antiporter functions using 10 helices, and an additional 2 contribute to assembly/stability. Proc. Natl. Acad. Sci. USA.

[18] Taglicht D, Padan E, Schuldiner S (1991). Overproduction and purification of a functional Na^+^/H^+^ antiporter coded by *nhaA* (*ant*) from *Escherichia coli*. J. Biol. Chem..

[19] Taglicht D, Padan E, Schuldiner S (1993). Proton-sodium stoichiometry of NhaA, an electrogenic antiporter from *Escherichia coli*. J. Biol. Chem..

[20] Dwivedi M, Sukenik S, Friedler A, Padan E (2016). The Ec-NhaA antiporter switches from antagonistic to synergistic antiport upon a single point mutation. Sci. Rep..

[21] Jardetzky O (1966). Simple allosteric model for membrane pumps. Nature.

[22] Masrati G (2018). Broad phylogenetic analysis of cation/proton antiporters reveals transport determinants. Nat. Commun..

[23] Inoue H, Noumi T, Tsuchiya T, Kanazawa H (1995). Essential aspartic acid residues, Asp-133, Asp-163 and Asp-164, in the transmembrane helices of a Na^+^/H^+^ antiporter (NhaA) from *Escherichia coli*. FEBS Lett..

[24] Noumi T, Inoue H, Sakurai T, Tsuchiya T, Kanazawa H (1997). Identification and characterization of functional residues in a Na^+^/H^+^ antiporter (NhaA) from *Escherichia coli* by random mutagenesis. J. Biochem..

[25] Galili L, Rothman A, Kozachkov L, Rimon A, Padan E (2002). Transmembrane domain IV is involved in ion transport activity and pH regulation of the NhaA-Na^+^/H^+^ antiporter of *Escherichia coli*. Biochemistry.

[26] Kozachkov L, Herz K, Padan E (2007). Functional and structural interactions of the transmembrane domain X of NhaA, Na^+^/H^+^ antiporter of *Escherichia coli*, at physiological pH. Biochemistry.

[27] Hu NJ, Iwata S, Cameron AD, Drew D (2011). Crystal structure of a bacterial homologue of the bile acid sodium symporter ASBT. Nature.

[28] Maes M, Rimon A, Kozachkov-Magrisso L, Friedler A, Padan E (2012). Revealing the ligand binding site of NhaA Na^+^/H^+^ antiporter and its pH dependence. J. Biol. Chem..

[29] Padan E, Schuldiner S (1993). Na^+^/H^+^ antiporters, molecular devices that couple the Na^+^ and H^+^ circulation in cells. J. Bioenerg. Biomembr..

[30] Rimon A, Mondal R, Friedler A, Padan E (2019). Cardiolipin is an optimal phospholipid for the assembly, stability, and proper functionality of the dimeric form of NhaA Na^+^/H^+^ antiporter. Sci. Rep..

[31] Gupta K (2017). The role of interfacial lipids in stabilizing membrane protein oligomers. Nature.

[32] Harel-Bronstein M (1995). MH1, a second-site revertant of an *Escherichia coli* mutant lacking Na^+^/H^+^ antiporters (*DnhaADnhaB*), regains Na^+^ resistance and a capacity to excrete Na^+^ in a Δμ̃_H_^+^-independent fashion. J. Biol. Chem..

[33] Ganea C, Fendler K (2009). Bacterial transporters: charge translocation and mechanism. Biochim. Biophys. Acta.

[34] Mager T, Rimon A, Padan E, Fendler K (2011). Transport mechanism and pH regulation of the Na^+^/H^+^ antiporter NhaA from *Escherichia coli*: an electrophysiological study. J. Biol. Chem..

[35] Huang Y, Chen W, Dotson DL, Beckstein O, Shen J (2016). Mechanism of pH-dependent activation of the sodium-proton antiporter NhaA. Nat. Commun..

[36] Quick M, Javitch JA (2007). Monitoring the function of membrane transport proteins in detergent-solubilized form. Proc. Natl. Acad. Sci. USA.

[37] Shi L, Quick M, Zhao Y, Weinstein H, Javitch JA (2008). The mechanism of a neurotransmitter:sodium symporter - inward release of Na^+^ and substrate is triggered by substrate in a second binding site. Mol. Cell.

[38] Zhao Y (2011). Substrate-modulated gating dynamics in a Na^+^-coupled neurotransmitter transporter homologue. Nature.

[39] Kantcheva AK (2013). Chloride binding site of neurotransmitter sodium symporters. Proc. Natl. Acad. Sci. USA.

[40] Stolzenberg S (2015). Mechanism of the association between Na^+^ binding and conformations at the intracellular gate in neurotransmitter:sodium symporters. J. Biol. Chem.

[41] LeVine MV (2019). The allosteric mechanism of substrate-specific transport in SLC6 is mediated by a volumetric sensor. Proc. Natl. Acad. Sci. USA.

[42] Olkhova E, Kozachkov L, Padan E, Michel H (2009). Combined computational and biochemical study reveals the importance of electrostatic interactions between the "pH sensor" and the cation binding site of the sodium/proton antiporter NhaA of *Escherichia coli*. Proteins.

[43] Shannon RD (1976). Revised effective ionic radii and systematic studies of interatomic distances in halides and chalcogenides. Acta Cryst..

[44] Cotton FA, Wilkinson G (1988). Advanced inorganic chemistry.

[45] Wohlert D, Kuhlbrandt W, Yildiz O (2014). Structure and substrate ion binding in the sodium/proton antiporter PaNhaP. Elife.

[46] Boudker O, Ryan RM, Yernool D, Shimamoto K, Gouaux E (2007). Coupling substrate and ion binding to extracellular gate of a sodium-dependent aspartate transporter. Nature.

[47] Lee C (2013). A two-domain elevator mechanism for sodium/proton antiport. Nature.

[48] Arkin IT (2007). Mechanism of Na^+^/H^+^ antiporting. Science.

[49] Rimon A, Dwivedi M, Friedler A, Padan E (2018). Asp133 rsidue in NhaA Na^+^/H^+^ antiporter is required for stability, cation binding and transport. J. Mol. Biol..

[50] Henderson JA, Huang Y, Beckstein O, Shen J (2020). Alternative proton-binding site and long-distance coupling in *Escherichia coli* sodium-proton antiporter NhaA. Proc. Natl. Acad. Sci. USA.

[51] Masrati G (2020). An angular motion of a conserved four-helix bundle facilitates alternating access transport in the TtNapA and EcNhaA transporters. Proc. Natl. Acad. Sci. USA.

[52] Mager T (2013). Differential effects of mutations on the transport properties of the Na^+^/H^+^ antiporter NhaA from *Escherichia coli*. J. Biol. Chem..

[53] Patino-Ruiz M (2019). Replacement of Lys-300 with a glutamine in the NhaA Na^+^/H^+^ antiporter of *Escherichia coli* yields a functional electrogenic transporter. J. Biol. Chem..

[54] Yamashita A, Singh SK, Kawate T, Jin Y, Gouaux E (2005). Crystal structure of a bacterial homologue of Na+/Cl–dependent neurotransmitter transporters. Nature.

[55] Rimon A, Tzubery T, Padan E (2007). Monomers of the NhaA Na^+^/H^+^ antiporter of *Escherichia coli* are fully functional yet dimers are beneficial under extreme stress conditions at alkaline pH in the presence of Na^+^ or Li^+^. J. Biol. Chem..

[56] Pinner E, Kotler Y, Padan E, Schuldiner S (1993). Physiological role of NhaB, a specific Na^+^/H^+^ antiporter in *Escherichia coli*. J. Biol. Chem..

[57] Rimon A, Kozachkov-Magrisso L, Padan E (2012). The unwound portion dividing helix IV of NhaA undergoes a conformational change at physiological pH and lines the cation passage. Biochemistry.

[58] Gerchman Y, Rimon A, Padan E (1999). A pH-dependent conformational change of NhaA Na^+^/H^+^ antiporter of *Escherichia coli* involves loop VIII-IX, plays a role in the pH response of the protein, and is maintained by the pure protein in dodecyl maltoside. J. Biol. Chem..

[59] Rimon A, Gerchman Y, Kariv Z, Padan E (1998). A point mutation (G338S) and its suppressor mutations affect both the pH response of the NhaA-Na^+^/H^+^ antiporter as well as the growth phenotype of *Escherichia coli*. J. Biol. Chem..

[60] Bradford MM (1976). A rapid and sensitive method for the quantitation of microgram quantities of protein utilizing the principle of protein-dye binding. Anal. Biochem..

